# Probabilistic mapping of the antiparkinsonian effects of pallidal deep brain stimulation

**DOI:** 10.1093/braincomms/fcaf374

**Published:** 2025-09-26

**Authors:** Pavel Navratil, Ghadir Abbas, Abdullah Elmas, Hazem F E Eldebakey, Anna-Sophie Schmidt, Vincent J Odekerken, Rob M A de Bie, ChenCheng Zhang, Katsuo Kimura, Robert Peach, Jonas Roothans, Jens Volkmann, Florian L Lange, Martin M Reich

**Affiliations:** Department of Neurology, University Hospital Wuerzburg, Wuerzburg 97080, Germany; Department of Neurology, University Hospital Wuerzburg, Wuerzburg 97080, Germany; Department of Neurology, University Hospital Wuerzburg, Wuerzburg 97080, Germany; Department of Neurology, University Hospital Wuerzburg, Wuerzburg 97080, Germany; Department of Neurology, University Hospital Wuerzburg, Wuerzburg 97080, Germany; Department of Neurology, Amsterdam University Medical Center, Amsterdam 1105 AZ, The Netherlands; Department of Neurology, Amsterdam University Medical Center, Amsterdam 1105 AZ, The Netherlands; Department of Neurosurgery, Shanghai Jiao Tong University, Ruijin Hospital, Shanghai 200025, China; Department of Neurology, Yokohama City University, Yokohama 236-0004, Japan; Department of Neurology, University Hospital Wuerzburg, Wuerzburg 97080, Germany; Department of Neurology, University Hospital Wuerzburg, Wuerzburg 97080, Germany; Department of Neurology, University Hospital Wuerzburg, Wuerzburg 97080, Germany; Department of Neurology, University Hospital Wuerzburg, Wuerzburg 97080, Germany; Department of Neurology, University Hospital Wuerzburg, Wuerzburg 97080, Germany

**Keywords:** deep brain stimulation, digital DBS programming, internal globus pallidus, Parkinson’s disease, probabilistic mapping

## Abstract

Deep brain stimulation of the internal globus pallidus effectively treats motor symptoms and drug-induced fluctuations in advanced Parkinson’s disease—but is complicated by a high variability in outcomes. In a minority of patients, even stimulation-induced aggravation of akinesia has been reported. Previous works and clinical practice suppose an antiparkinsonian sweetspot in a sensorimotor segment in ventrocaudal internal globus pallidus. However, the detailed functional anatomy of the nucleus is still a matter of debate. We examined 39 patients with Parkinson’s disease undergoing pallidal deep brain stimulation from two centres. Deep brain stimulation outcomes were scaled using the percentage change in the motor part of Unified Parkinson’s Disease Rating Scale in MED-OFF pre- and postoperatively. The mean improvement with chronic pallidal deep brain stimulation was 16 ± 37%. Motor symptoms improved in 72% of patients. However, 10% were poor responders (<15% improvement) and 28% experienced a worsening of parkinsonism. After basic data processing, we used individual stimulation parameters to construct volumes of tissue activated and applied an established approach of voxelwise *t*-statistic to build a probabilistic map of the antiparkinsonian effect of deep brain stimulation in the internal globus pallidus. Subsequently, we sampled the volumes of tissue activated in a leave-one-out fashion and employed a multivariate linear regression model to predict an individual outcome. Using this framework, we explained 72% of the variance in motor outcomes (*P* < 0.001) by the spatial effect map. The linear model was significantly predictive in the leave-one-out cross-validation (Pearson’s *R* = 0.62; *P* < 0.001). The pallidal subregion providing the best antiparkinsonian effect—as defined by the most significant voxels of the probabilistic map—was located anteriorly in the posteroventral internal globus pallidus. Furthermore, we used the probabilistic map to simulate a monopolar review in silico in each patient and calculated optimized stimulation parameters, demonstrating the potential use of our model in image-guided programming.

## Teaser

Navratil and colleagues used computer modelling and advanced image processing to describe the functional anatomy of the pallidal complex in the context of deep brain stimulation. They created a comprehensive map of the region, predicted clinical outcomes based on stimulation volumes, and simulated optimized stimulation settings for each patient.

## Graphical Abstract

**Graphical Abstract:**
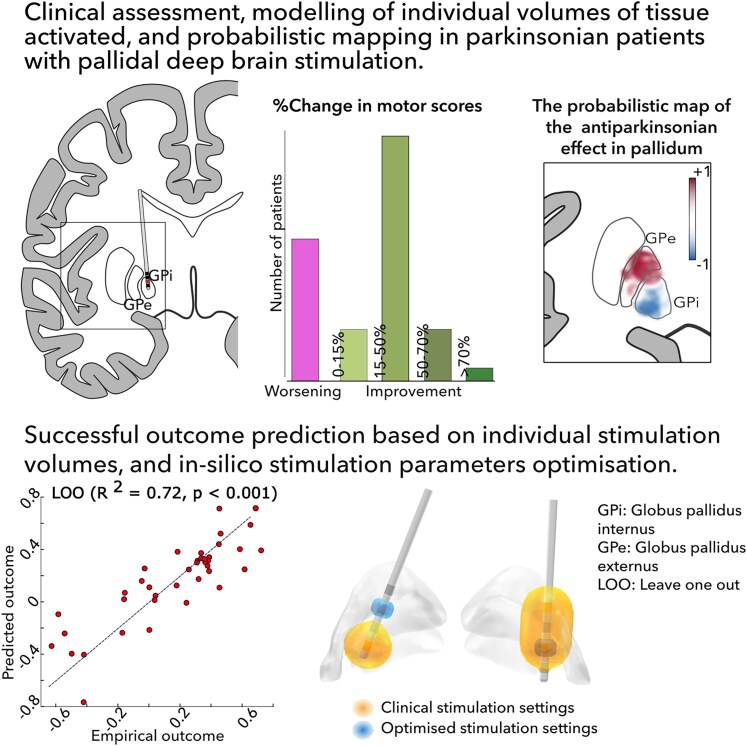

## Introduction

Historically, pallidotomy was successfully used to treat patients with advanced Parkinson’s disease. When applied unilaterally, the procedure effectively reduced akinesia, rigidity, and L-DOPA-induced dyskinesias with a low-risk profile.^1^

Today, deep brain stimulation (DBS) dominates the neurosurgical treatment of Parkinson’s disease, offering further benefits of reversibility and postoperative adjustments and potentially allowing a symptom-specific connectomic treatment approach.^2^ DBS of the internal globus pallidus (GPi) and the subthalamic nucleus (STN) are now standard-of-care treatments for advanced Parkinson’s disease.^3-5^ In Europe, STN-DBS has been the predominant choice for alleviating motor symptoms in Parkinson’s disease due to its more pronounced effect on off-period motor symptoms and the potential to reduce dopaminergic medication.^4,6-8^ However, GPi-DBS is considered to have fewer risks of cognitive decline, gait disturbances, and postoperative dyskinesias—rendering it a valuable alternative, especially for elderly or frail patients who might be at higher risk of STN-DBS-associated side effects.^8-10^ Having the choice of two surgical target regions enlarges the pallet of therapeutical tools, increasing the pool of patients eligible for DBS. In contrast to Europe, many centres in North America use the GPi as the primary preferred target,^8^ but typically also operate on an older patient population.

Despite its merits, the reported variability in GPi-DBS outcomes is significant, with some patients even experiencing a worsening of motor symptoms in the MED-OFF state.^11^ This is in line with observations of parkinsonian symptoms induced by chronic pallidal stimulation in dystonia patients. Early works have proposed a functional segregation of GPi into a ventral antidyskinetic and a more dorsal antiparkinsonian region.^12,13^ In these studies, stimulation of proximal contacts of the quadrupolar lead was associated with better reduction of akinesia, whereas acute stimulation of the distal contacts could worsen akinesia but reduce L-DOPA-induced dyskinesias. However, these studies were ‘lead centric’ and did not account for the underlying anatomy. In fact, it was strongly debated whether the dorsal ‘antiparkinsonian’ region was still within GPi, the border zone of the GPi and external globus pallidus (GPe), or the GPe proper. Later, an antiparkinsonian sweetspot was localized to the posteroventral GPi in a study with 28 subjects.^14^ Using a connectivity-driven segmentation, Middlebrooks and colleagues^15^ described an association between clinical improvement of Parkinsonism and stimulation of the M1-connected GPi segment, lying in the posteroventral part of the nucleus. Induced dyskinesias^16,17^ as well as worsening of bradykinesia^11^ have been related to dorsal stimulation at the GPe border. The posteroventral (ventrocaudal), sensorimotor territory of the GPi has been established as the optimal stimulation target. However, a detailed, comprehensive map of the pallidal region enabling a reliable clinical outcome prediction is still missing, and the pallidal functional anatomy remains a matter of discussion in the scientific and clinical DBS community, especially in the context of high outcome variability.

In our study, we attempted a probabilistic mapping of the antiparkinsonian effects of GPi-DBS. By examining a cohort of patients from two independent centres, we strived to define the optimal stimulation volumes within the pallidal region, or more specifically, in the subregion of ventrocaudal GPi, which has been considered the optimal implantation target based on previous literature. Our approach used a common normative space and anatomical atlases to precisely localize DBS effects, enabling a systematic analysis of larger patient cohorts. We integrated advanced modelling of stimulation fields and voxelwise statistics to provide a detailed anatomical basis for the observed clinical outcomes. Our ultimate goal was to design observer-independent, image-guided tools for the programming of pallidal DBS in PD.

## Materials and methods

### Subjects

We retrospectively enrolled patients diagnosed with idiopathic Parkinson’s disease treated with bilateral GPi-DBS from DBS centres in Shanghai, China, and Amsterdam, The Netherlands. The Amsterdam data were collected within a randomized controlled trial.^4^ All patients were screened for the availability of pre- and postoperative motor scores (Amsterdam cohort: Unified Parkinson’s Disease Rating Scale (UPDRS) III; Shanghai cohort: Movement Disorder Society (MDS)-UPDRS III) in the MED-OFF state and a sufficient quality of imaging data; in all patients, preoperative isotropic T1 MRI sequence with resolution < 1 mm and postoperative CT or MRI were required. The quality was assessed manually by two experienced DBS neurologists regarding atlas segmentation and electrode localization. The images automatically recognized as insufficient for the Lead DBS pipeline^18^ were also excluded.

The data collection was carried out in accordance with the 1975 Declaration of Helsinki.

### Surgical procedure and clinical evaluation

Posteroventral GPi was the surgical target in all patients. All subjects were implanted with quadripolar non-directional leads (Medtronic 3389, Medtronic 3387, SceneRay 1210, PINS L 302). MDS-UPDRS III or UPDRS III scores were acquired as part of the presurgical work-up, and the same scores were taken 6–28 months after surgery under chronic stimulation. All MED-OFF clinical data were collected after overnight withdrawal of the dopaminergic medication. We calculated the percentage improvement in motor scores. To classify the clinical response of patients after DBS surgery, we grouped improvements in motor scores into five categories: Excellent response: > 70% score reduction; Good response: 50–70% score reduction; Medium response: 15% to 50% score reduction; Non-responders: 0–15% score reduction; Worsening: any percentage increase in score.

**Figure 1:**
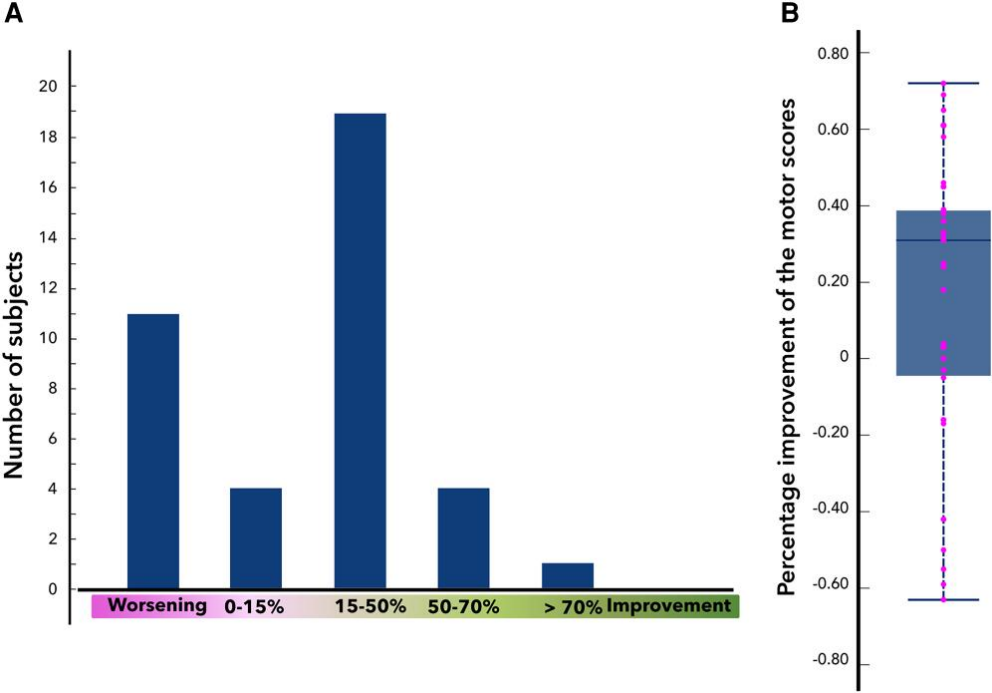
**Distributions of MED-OFF empirical clinical outcomes (percentage change of UDPRS III or MDS-UPDRS III) as histogram (A) and boxplot (B).** Individual data points represent single subjects (**B**). Note the high prevalence of symptom worsening (28%).

The use of different UPDRS motor scales was accounted for by calculating the proportional score changes for each individual. To present a synoptical overview of the baseline scores as a mean and a standard deviation in the whole cohort (Fig. 1), we calculated a mean UPDRS III score per single item (a normalized score) in each Amsterdam patient and rescaled it to MDS-UPDRS III by multiplying by the number of its items (33).

We also performed an exploratory subcohort analysis of the GPi-DBS on-medication effects, using available Amsterdam MED-ON data. We calculated the percentage reductions in UPDRS III in MED-ON, the levodopa-equivalent dose (LED), and the clinician-based clinical dyskinesia rating scale (CDRS), analogous to the percentage reduction in UPDRS III in MED-OFF.

### Statistical analysis

The statistical approaches used to generate the heatmap and build the imaging-based predictive models are described in detail in the following section below. We used the sample mean ± standard deviation, where applicable, as descriptive statistics for continuous variables. Categorical variables were summarized with the number and percentage of patients. To assess the significance of paired sample differences, we applied two-tailed, or when testing unidirectional inequality, one-tailed, Wilcoxon signed-rank tests. To test for dependencies between AC-sweetspot distances, stimulation parameters, and clinical outcomes, a multivariate linear model was employed.

### Imaging data processing, DBS lead localization, and stimulation field modelling

To obtain models of stimulation fields in the normative space, enabling statistical analysis on a group level, we followed the processing steps thoroughly described in an earlier study.^19^ We applied the software SureTune3 (Medtronic) to linearly coregister the acquisitions (pre- and postoperative MRI and CT scans). We performed semiautomatic tissue segmentation and manual lead localization as implemented in SureTune3. The stimulation fields were modelled as volumes of tissue activated (VTAs), employing Astrom’s VTA model.^20^ This VTA model considers, in addition to amplitudes, also varying pulse widths and is based on computational models of axon activation. Stimulation parameters at the time of the postoperative clinical assessment were used to model the VTAs.

The sessions, consisting of leads, VTAs, and segmented GPis in the native space, were exported from SureTune3. Finally, we used the custom MATLAB toolbox Arena to transform the VTAs to the normative space (MNI_ICBM_2009b_NLIN_ASYM).^21^ The DISTAL atlas^22^ (in the normative space) and the SureTune data (native space) were imported into Arena. In the following step, the individually segmented GPis from SureTune were linearly reregistered to the atlas, maintaining the relative electrode and VTA position in the nuclei. To enable an analysis of all data within a single GPi model, all VTAs from the right hemisphere were mirrored to the left. At least two experienced neurologists inspected all steps manually.

### Voxelwise probabilistic mapping and prediction of the stimulation outcome

All pairs of VTAs were labelled by the percentage motor score change. Each voxel in the target region was covered by multiple VTAs; therefore, an outcome distribution could be assigned to each voxel. We performed a two-sample *t*-test of the outcomes assigned to each voxel against the rest of the clinical outcomes, which led to a *t*-map and a p-map. We constructed a signed p-map (also called a heatmap) as 1-p multiplied by the sign of the *t*-value, reflecting the direction of the statistical relationship (showing if the voxel was rather related to a score increase or decrease). The heatmap values ranged from −1 for the most detrimental voxels to +1 for the most beneficial voxels. Sweetspots and sourspots were visualized as clusters (>50) of significant (*P* < 0.05) voxels associated with good or poor outcomes, respectively. Smoothing with a 3-voxel kernel, implemented in MATLAB, was applied to the resulting 3D shapes.

In the next step, we intended to estimate how the precision of sweetspot targeting predetermines a clinical outcome. We calculated the centrum of gravity (COG)—defined as the average coordinate of a 3D object - of the sweetspot and its distance from each active contact. We used a univariate linear regression model to test for a relationship between the mean distance of a patient’s active contacts to the sweetspot’s COG and the patient’s clinical outcome.

Subsequently, we trained a multivariate linear regression model to forecast the clinical outcome based on interference histograms. To avoid circularity and maintain the independence of the training data, we proceeded in a leave-one-out (LOO) fashion. After leaving a subject out, the heatmap was recalculated from *n*-1 subjects. The two VTAs of the left-out subject were sampled on the heatmap. Fifteen bins interference histograms, representing the distribution of the heatmap values inside the VTAs, were created. This process was repeated *n* times, resulting in *n* heatmaps and *n* independent histograms. Finally, a multivariate linear model with 15 predictors (bins of the histograms) and empirical improvement as the response variable was fitted. In other words, the range of the heatmap (−1 to +1) was divided into 15 bins (≅0.13 long steps); counts of the voxels encompassed by each VTA, falling into particular bins, were used as predictors. We cross-validated the model in the LOO fashion (LOO cross-validation, LOOCV). In this step, we used the set of *n* independent histograms created previously. In each iteration, we trained the model on *n*-1 histograms and used it to predict the empirical improvement of the left-out subject. Following this, we calculated Pearson’s correlation between the predicted value and the empirical improvement. To further validate the heatmap, and to control for overfitting another way, we created nine bins interference histograms and fitted a linear model with nine predictors. Because of the smaller sample size, we primarily applied the nine bin histograms in the subcohort analysis of GPi-DBS on-medication effects.

### Validation with lead DBS and lead group

To facilitate the robustness of our conclusions with a different methodological framework, we used the Lead DBS^18^ and Lead Group^23^ toolboxes to process the data and model the stimulation fields according to the pipeline described by Horn *et al.*^24^ We visually inspected the electrode scenes in both Arena and Lead Group and checked that the anatomical positions of the leads matched. We performed voxelwise mapping using Spearman’s correlation of the E-fields’ magnitudes and empirical improvements. For a detailed argumentation for the choice of this statistical procedure, see Horn *et al.*^24^ We validated the R-map by predicting the empirical improvements as the mean of the R values encompassed by each pair of the VTAs in an LOO fashion. Finally, we visually compared the R-map constructed in Lead Group and the heatmap constructed in Arena and calculated their Pearson’s spatial correlation for similarity check.

### DBS programming in silico

We aimed to facilitate DBS programming by digitally predicting optimal stimulation parameters. To demonstrate this application of our model, we again used a similar approach to Reich *et al.*^19^ We applied Arena to visualize all leads in the MNI space. We simulated a monopolar review in all patients by predicting possible clinical outcomes for every combination of standard VTAs, applying our multivariate linear regression model and our heatmap. For each contact, we tested VTAs using a set pulse width (60 μs) and varying amplitudes (1, 2, 3, 4 and 5 mA), resulting in a set of theoretical programming choices that were ranked by predicted outcomes.

## Results

### Clinical characteristics

Sixty patients with idiopathic Parkinson’s disease treated with bilateral GPi-DBS were enrolled from Shanghai (*n* = 33) and Amsterdam (*n* = 27). Fifty four patients had complete clinical datasets, but 15 were excluded due to insufficient imaging data quality, resulting in a final study cohort of 39 patients (Shanghai, *n* = 16; Amsterdam, *n* = 23). Of these, 15/39 were female and the mean age at surgery was 62 ± 6 years. The mean baseline MDS-UPDRS III (rescaled from UPDRS III for the Amsterdam cohort) was 53 ± 17 points, and the mean time from surgery to the clinical evaluation was 12 ± 3 months.

The MED-OFF clinical outcome of DBS in our cohort was highly variable. The mean improvement was 16 ± 37%. One patient (2%) had an off-period motor score reduction >70% (excellent response), four (10%) had a reduction of 50–70% (good response), 19 (49%) had a reduction of 15–50% (medium response), four (10%) were non-responders (score reduction <15%), and 11 (28%) had an increase in motor score (worsening) (Fig. 1). The symptoms in MED-OFF were significantly improved at the postop follow-up; the median percentage improvement was positive with *P* = 0.02 and the normalized absolute scores decreased postoperatively with *P* < 0.001.

**Figure 2:**
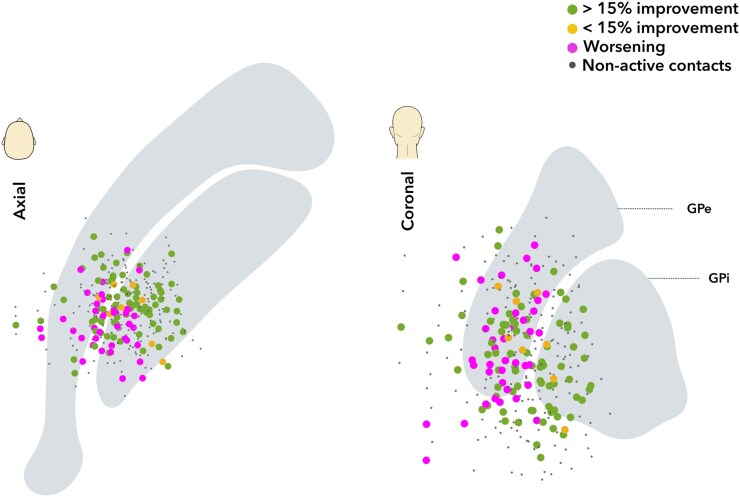
**Lead contacts projected on the coronal and axial plane.** Each data point represents a single DBS contact. The active contacts covered the posterior part of the pallidal complex. Active contacts of responders (>15%) are in green, non-responders (<15%) in yellow, and those of patients with worsening in magenta. Non-active contacts are depicted as small black dots. We found only a weak relationship between the distance of active contact to the sweetspot and clinical outcome, highlighting the need for a more advanced method.

The mean stimulation frequency was 126 ± 32 Hz (median 130 Hz) and the mean pulse width was 77 ± 32 μs (median 60 μs). All patients were stimulated using a constant voltage (mean 3 ± 0.5 V). One patient had bipolar stimulation, all other patients had cathodic stimulation. The stimulation parameters did not significantly explain the variance in clinical outcome (*R*^2^ = 0.23, *P* = 0.19) in a multivariate linear regression model. The lead contacts were spread in the posterior part of the pallidal complex, extending from the white matter adjacent to the medial GPi to the medial putamen, and from the subpallidal white matter to the dorsal GPe (see Fig. 2).

In our Amsterdam subcohort, the DBS clinical outcomes in MED-OFF were similar to the outcomes in the full cohort. The mean percentage reduction in UPDRS III in MED-OFF after GPi-DBS was 17 ± 39%. In MED-ON (from preoperative MED-ON to postoperative MED-ON, STIM ON), the mean percentage reduction was −8 ± 62%, showing even higher variability than the OFF data and a slight increase in UPDRS III on the group level. The mean percentage reduction in LED after GPi-DBS was 20 ± 23%. Dyskinesias measured with CDRS showed a decrease of 50 ± 61%. The median LED percentage reduction was greater than zero (*P* < 0.001), and the median CDRS reduction was greater than zero (*P* = 0.002); these findings indicated that after GPi-DBS, both LED and dyskinesia had significantly decreased.

In the Shanghai cohort alone, the mean MDS-UPDRS III percentage improvement (in MED-OFF) was 14 ± 35%.

See the Supplementary Table 1 (the cohort summary) for the complete clinical data.

### Voxelwise probabilistic mapping and prediction of the stimulation outcome

**Figure 3:**
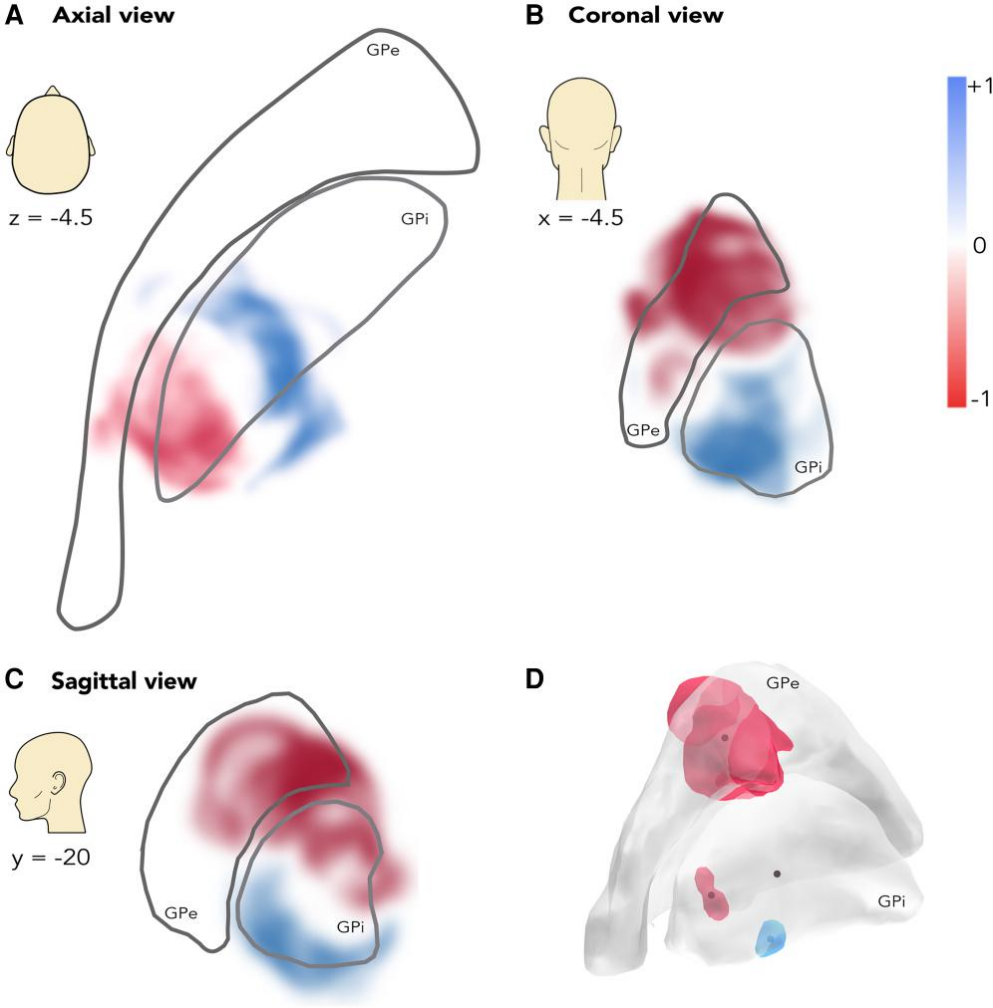
**The antiparkinsonian heatmap of the pallidal region in axial (A), coronal (B), and sagittal slices (C) was constructed using voxel-wise two-sample *t*-tests, comparing outcomes assigned to each voxel against the remaining clinical outcomes within the whole cohort (*n* = 39).** The resulting signed p-map (heatmap) is displayed. Detrimental voxels are depicted in tones of red, and beneficial voxels are in tones of blue. The heatmap was smoothed with a 3-voxel kernel for visualization. The clusters (>50) of significant voxels (*P* < 0.05) formed sweetspots and sourspots within the pallidal complex in 3D, the COGs are depicted as points **(D)**. See also Supplementary Fig. 7 for comparison with the 2019 antidystonic heatmap.^19^.

Our heatmap of the antiparkinsonian effect of GPi-DBS ((MDS-)UPDRS III reduction in MED-OFF) showed a gradient in the dorsal to ventral and posterolateral to anteromedial directions, with beneficial regions located ventrally in the central GPi (Fig. 3A–C) When applying the threshold of *P* < 0.05, we found a sweetspot located at the ventromedial edge of the GPi, slightly posterior to the geometric centre of the nucleus in the anteroposterior axis (MNI coordinates of the COG: −18.4, −7.3, −7.0; in relation to the midcommissural point coordinates in the MNI template: −18.4, 4.5, −3.7). The sourspots—related to a worsening of off-period motor symptoms—encompassed an area in the central GPe (dorsal to the sweetspot) and a smaller area in the GPi (dorsal and caudal to the sweetspot) (Fig. 3D). The mean distance from the sweetspot COG was significantly associated with the clinical outcome (*R*^2^ = 0.22, *P* = 0.01).

**Figure 4:**
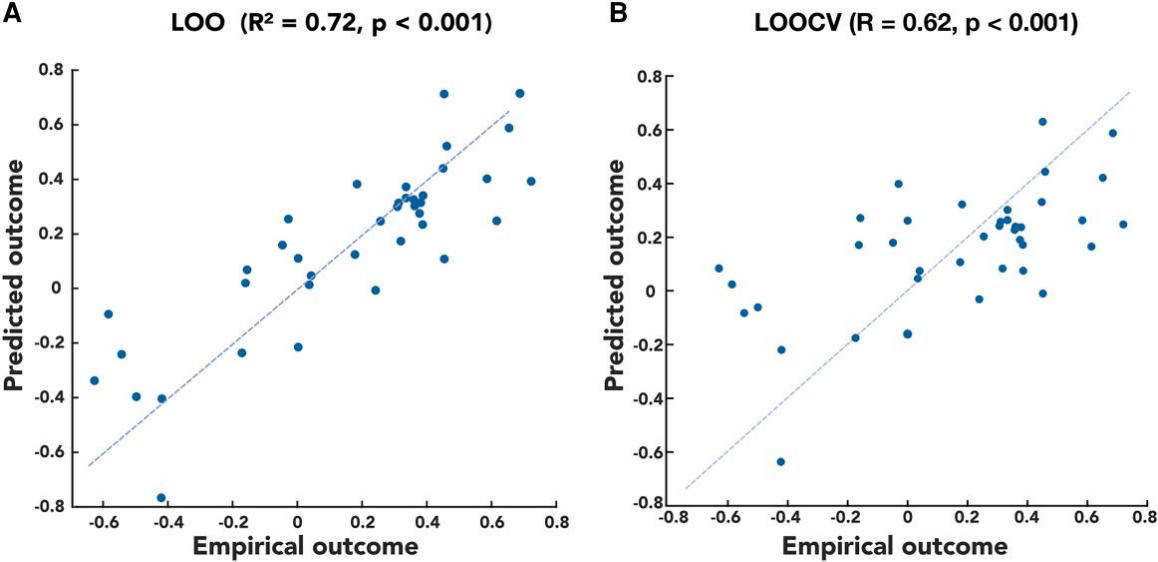
**The multivariate linear model with data points representing single subjects.** Interference histograms acquired in LOO settings were used as input; the model explained 72% of the outcome variance (**A**) and the *F*-test revealed high significance (*P* < 0.001). Simulating a prediction of an unseen patient in LOOCV showed a high Pearson’s correlation of empirical and predicted outcomes (*R* = 0.62, *P* < 0.001) (**B**). LOO(CV), LOO (cross-validation).

The multivariate linear regression model—employing the interference histograms obtained in the LOO fashion, predicting off-period motor score changes—showed an excellent fit (*R*^2^ = 0.72, *P* < 0.001); it explained 72% of the outcome variance in our sample. After reducing the number of predictors to nine, the fit remained excellent (*R*^2^ = 0.57, *P* < 0.001). The LOOCV (retraining the linear model in each iteration to further control for overfitting) showed a high validity of the model (Pearson’s *R* = 0.62, *P* < 0.001) (Fig. 4). The R-map, resulting from the validation step in the Lead Group, was also significantly predictive in the LOO fashion (Pearson’s *R* = 0.38, *P* = 0.014) (Supplementary Figs 1 and 2). When visually checked, the two maps showed the same spatial gradients and were significantly spatially correlated, although with relatively low R (Pearson’s *R* = 0.26, *P* < 0.001).

### Exploratory analysis of GPi-DBS MED-ON effects in the Amsterdam subcohort

**Figure 5:**
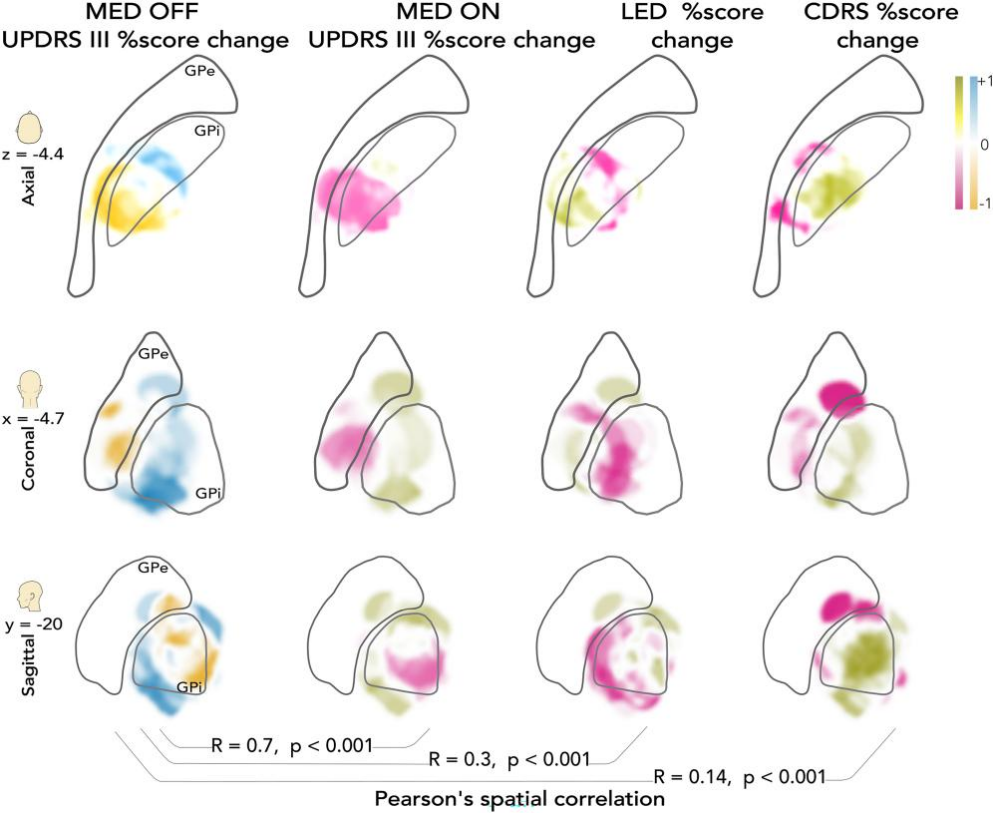
**The heatmaps comparison of the MED-OFF antiparkinsonian heatmap with the (i) MED-ON antiparkinsonian heatmap (second column), (ii) LED reduction heatmap (third column), (iii) CDRS reduction heatmap (fourth column).** The heatmaps are presented in axial, coronal, and sagittal slices. Voxels are depicted in tones of orange (detrimental) and blue (beneficial) for the MED-OFF map, and as magenta (detrimental) and olive green (beneficial) for other maps. The Pearson’s spatial correlation coefficients of the MED-OFF map with the MED-ON, LED reduction, and CDRS reduction maps were *R* = 0.7, *P* < 0.001, *R* = 0.3, *P* < 0.001, and *R* = 0.14, *P* < 0.001, respectively. All maps were calculated from the Amsterdam cohort (*n* = 23). CDRS, clinical dyskinesia rating scale; LED, levodopa-equivalent dose; UPDRS, Unified Parkinson’s Disease Rating Scale.

First, we recalculated the MED-OFF UPDRS III reduction heatmap solely from the Amsterdam data. This map showed an analogous spatial distribution to the full cohort map. In the following steps, we created MED-ON maps for the UPDRS III score change, LED change, and CDRS change, and compared them with the MED-OFF UPDRS III change map (Fig. 5). These maps were not consistently predictive (for details, see Supplementary Figs 3–6).

As expected, the MED-ON and MED-OFF UPDRS III change maps were highly similar (Pearson’s *R* = 0.7, *P* < 0.001). The LED change map also showed an analogous spatial distribution, with areas of the best medication reduction located near the antiparkinsonian sweetspot; although relatively lowly, it was spatially correlated with the MED-OFF map (Pearson’s *R* = 0.3, *P* < 0.001). Finally, the CDRS reduction map exhibited wide areas with dyskinesia reduction in the central GPi, partially overlapping with the sweetspot, but also the more posterior sourspot of the off-period motor score change. The two maps were correlated with a low correlation coefficient (Pearson’s *R* = 0.14, *P* < 0.001) (Fig. 5).

### DBS programming in silico

**Figure 6:**
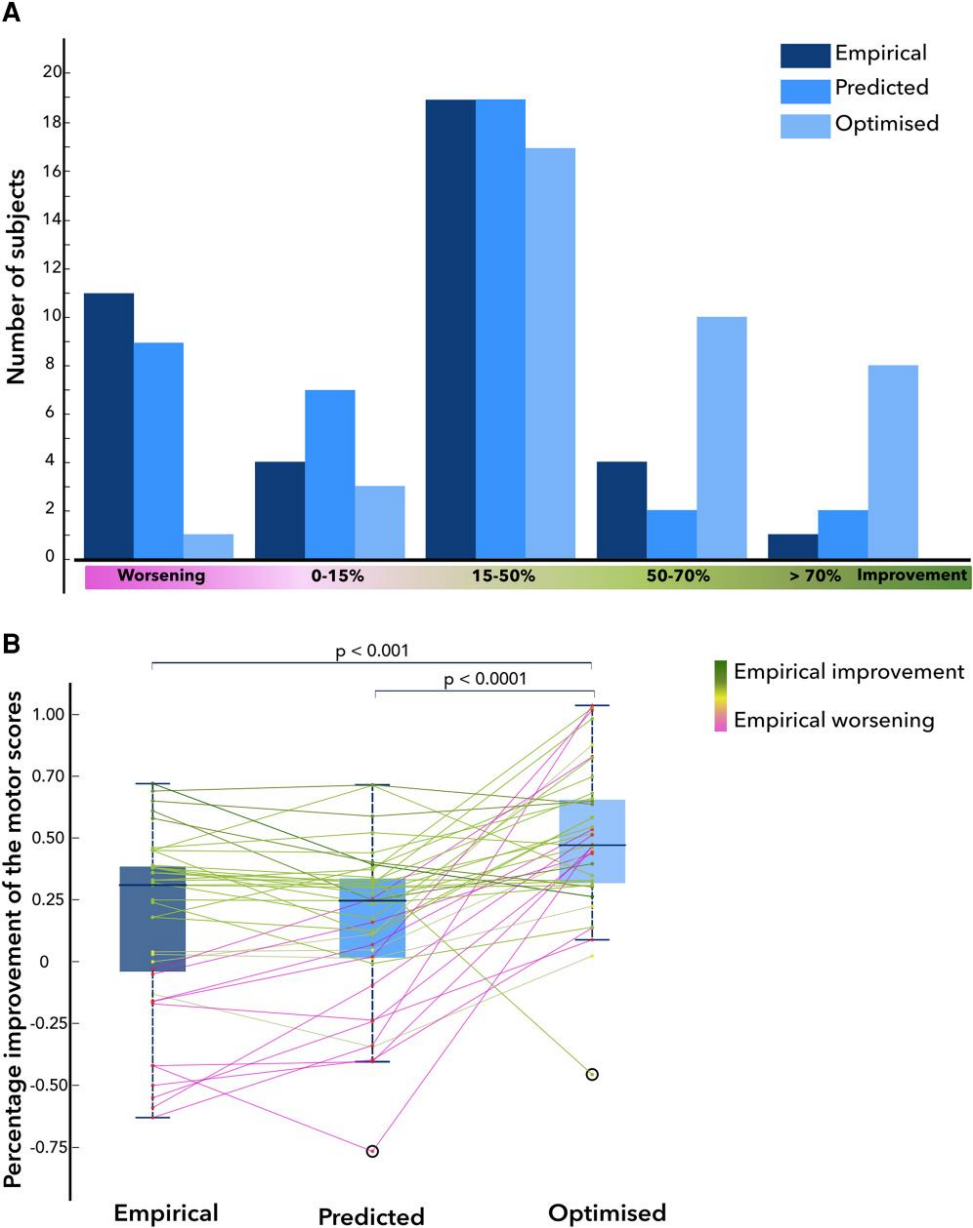
**Distributions of empirical, predicted, and optimized clinical outcomes as histogram (A) and boxplots (B) in the whole cohort (*n* = 39).** Individual subjects are represented as data points connected with lines (**B**). Tailed Wilcoxon signed-rank test revealed a significant superiority of optimized outcomes over empirical outcomes (*P* < 0.001) and of optimized outcomes over predicted outcomes (*P* < 0.0001). After the digital optimization, only a single patient had a hypothetical worsening based on the algorithmic programming choice, suggesting a suboptimal lead localization in this subject.

To show the possible clinical use of the probabilistic outcome mapping, we suggested optimized stimulation parameters for all patients in our cohort, based on the top ranking of all simulated VTAs (see Materials and Methods). The mean amplitude of the optimized settings was 1.7 ± 1.2 mA (the mean clinical voltage was 3.0 ± 0.5 V). The mean optimized outcome was 50 ± 30%. Only a single patient had a predicted worsening based on the algorithmic programming choice (Fig. 6). Using the tailed Wilcoxon sign rank test and 5% as the minimal clinically meaningful difference, we showed the superiority of the optimized outcomes over empirical outcomes (*P* < 0.001) and over predicted outcomes based on the actual clinical settings (*P* < 0.0001). However, the predicted and optimized outcomes are *simulated* values that cannot be reliably compared with the empirical measurements (although trained on empirical data).

**Figure 7:**
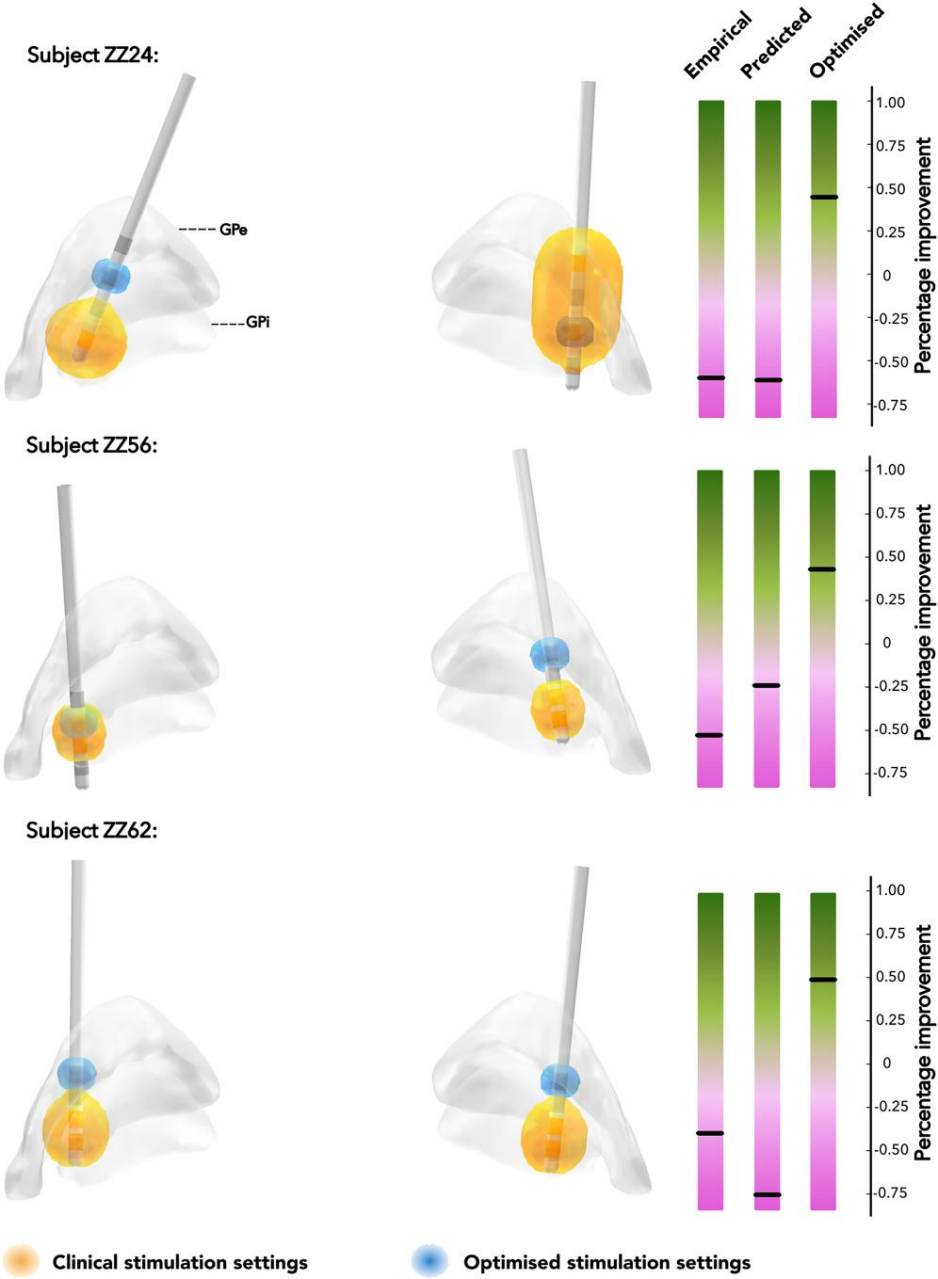
**Demonstration of digital programming troubleshooting in three patients with worsening of off-period motor symptoms after DBS.** Clinical volumes of tissue activated (VTAs) are in orange, the optimized algorithm-predicted VTAs are in blue. Note the non-trivial nature of the algorithmic solutions. The optimized VTAs were not consistently shifted towards the group sweetspot. This is a consequence of the rather complex 3D shape of the heatmap, with local minima and maxima. This phenomenon highlights the advantage of algorithmic programming using a probabilistic outcome map over image-guided programming, by visually shaping the VTA to a sweetspot volume. The optimized VTAs led to a substantially better simulated outcome compared to the clinically observed outcome. Of note, the stimulation energy for optimized VTAs was lower compared to the clinical stimulation parameters.

Furthermore, we demonstrated digital troubleshooting by selecting three subjects with empirical worsening who would potentially have a good improvement if the digitally predicted parameters were used (Fig. 7).

## Discussion

In patients with Parkinson’s disease treated by GPi-DBS, clinical follow-up showed a high variability in motor outcomes. Employing probabilistic outcome mapping,^19^ we were able to explain a large part of the variance in off-period motor symptom change (72%) by the anatomical location and the extent of the stimulated tissue. This result confirms that the improvement in disease motor symptoms in Parkinson’s disease using pallidal DBS critically depends on the lead placement and optimal programming choice for the stimulation volume.

### Clinical variability in pallidal DBS outcomes in Parkinson’s disease

Comparative studies have found greater mean off-period motor score improvements with STN-DBS than with GPi-DBS.^4,7,8^ This may be partially related to the high variability in outcome with GPi-DBS for Parkinson’s disease in MED-OFF, as reported by Sobesky *et al.*^11^; they observed a mean improvement of 13%, with a coefficient of variation of 323% (SD 42%). The Amsterdam cohort in our study partially overlapped with the patient group in Sobesky *et al.*^11^; after grouping with the Shanghai cohort, our overall mean improvement was 16 ± 37%. In the STN-DBS cohort of Sobesky *et al.,*^11^ however, the coefficient of variation was much lower at only 49.8% (mean improvement 47 ± 23%).

Okun and Foote^25^ proposed that the larger size of the GPi compared to the STN may account for less consistent lead placement and more variable outcomes. Moreover, clinical programming of STN-DBS is guided by typical extrinsic side effects of stimulation in addition to acute antiparkinsonian effects^26^; whereas in the GPi, only the ventral and medial borders are electrophysiologically eloquent,^27^ rendering clinically guided programming more difficult. Hence, outcome maps of the effects of pallidal stimulation could potentially help to leverage the results for STN-DBS by more consistent planning of the pallidal stimulation volume—which was indeed suggested by our in silico simulated programming optimization.

### Topography of the antiparkinsonian effects

We found the antiparkinsonian sweetspot within the ventromedial portion of the GPi, slightly posterior to the centre of the nucleus in the longitudinal axis (Fig. 3D). The beneficial areas of the heatmap extended centrally along the GPi’s ventral border (Fig. 3). The sensorimotor GPi, supposedly located posteroventrally, was traditionally considered the optimal DBS-target in Parkinson’s disease^28^ and dystonia.^29,30^ Of note, dorsal stimulation in the GPe and at its caudal border was predominantly detrimental for off-period motor symptoms (Fig. 3). In contrast to our findings, Krack *et al.*^13^ described an acute worsening of akinesia and a reversal of the levodopa response by stimulating the most ventral electrode contacts in the pallidal region. These contacts, however, were effective in reducing other off-period parkinsonian symptoms (such as rigidity and freezing), as well as on-dyskinesia. Based on this early study, the concept of the ‘2-D GPi triangle’ with two functional zones was established.^17^ A possible explanation for the discrepancy between ours and Krack’s observations may be the lack of a clear anatomical association for the observed stimulation responses. Krack *et al.*^13^ examined finger tapping speed and amplitude as a marker of bradykinesia—but subliminal tetanic stimulation of the internal capsule may diminish alternating movement speed and amplitude, thereby mimicking a pro-akinetic effect. Sobesky *et al.*^11^ reported bradykinesia worsening in patients with Parkinson’s disease treated with GPi-DBS, associated with stimulation fields reaching into the dorsal, posterior zone at the border with GPe. This area aligns with our second, smaller sourspot (Fig. 3).

The traditional 2-D perception of GPi was challenged by the notion that suboptimal electrode location in the anteroposterior and mediolateral axes also significantly influences clinical outcomes.^17^ Middelbrooks *et al*.^15^ segmented the nucleus based on the connectivity profiles and found an association between stimulation of the M1-connected segment in the posterior GPi and clinical improvement. Elias and colleagues^14^ performed probabilistic mapping on a smaller cohort of 28 patients and found a sweetspot in ventrocaudal GPi. Of note, the authors used a rather ‘hotspot’ oriented approach, identifying significant voxel clusters for each DBS indication. Our aim was, in contrast, to create a fine, comprehensive map of the pallidal region that predicts an individual clinical outcome as a unit. Closer comparison shows Elias’s sweetspot extending posteriorly to the nucleus’ border, overlapping with areas related to suboptimal outcomes in our map. Also, our sweetspot’s COG is located slightly medial and anterior to that of Elias, favouring boldly more central stimulation in the longitudinal axis, albeit still somewhat posterior to the GPi’s geometric centre.

Despite fundamental methodological differences, visual inspection of the R-map created in the Lead Group^23^ showed roughly the same spatial gradients (caudorostral along the axis of the GPi, dorsoventral and lateromedial) (Supplementary Fig. 1). The major differences were in the basic data processing (normalization into MNI space, brain shift correction, see Horn *et al.*^18^ for details), modelling of the stimulation fields, and in the statistics used in single voxels (see Horn *et al.*^24^ for details about E-field modelling and R-map creation). The maps were significantly spatially correlated, despite the relatively lower spatial Person’s R value.

Interestingly, the gradients of the previously published antidystonic heatmap^19^ and our antiparkinsonian heatmap seem perpendicular in the axial plane (Supplementary Fig. 7). The sign of the antidystonic heatmap changed along the mediolateral axes, whereas the sign of the antiparkinsonian heatmap reversed predominantly in the direction of the longitudinal axes of the nuclei. This phenomenon could be explained by varying fibre tracts mediating improvement and worsening in the two DBS indications, and should motivate further research using connectomic methods. Horn *et al.*^24^ described a differentiation of pallidothalamic projection subsystems participating in improvement in GPi-DBS for cervical and generalized dystonia. Should the fasciculus and ansa lenticularis (pallidothalamic projections) play a major role in dystonia improvement, it is possible to hypothesize that the Edinger comb (pallidosubthalamic projection)—an important connection within the indirect pathway—could be of greater importance in mediating antiparkinsonian effects, as recently suggested by connectomics.^2^ The partially perpendicular orientation of these fibre systems in the GPi could explain the perpendicular gradients of the disease-specific heatmaps. This hypothesis is further supported by poor outcomes of stimulation through proximal lead contacts in STN-DBS,^31^ which may activate pallidothalamic fibres alone but miss the Edinger comb and hyperdirect pathway.

Despite the different heatmap gradients, the antidystonic and antiparkinsonian sweetspots lie in a tight vicinity. This suggests a single ideal lead implant target within the anterior part of the ventrocaudal GPi for both indications (Supplementary Fig. 7). Our results, however, suggest that improved steering capability within the target region and optimized shaping of the VTA could further improve outcomes.

### Exploratory analysis of the effects of GPi-DBS MED-ON in the Amsterdam subcohort

We explored probabilistic mapping with the MED-ON data available in our Amsterdam cohort. Despite the smaller sample size and lack of predictiveness in the validation steps, this analysis brought first insights into the anatomical bases of the clinically relevant interaction between the GPi-DBS and dopaminergic medication.

The MED-OFF and MED-ON UPDRS III reduction heatmaps exhibited similarity. Consistently with the assumption that reducing dopaminergic medication is enabled by the direct antiparkinsonian effect of DBS, the LED reduction heatmap also showed a congruent spatial distribution (Fig. 5). The minor differences between these maps may have resulted from other factors and limitations of our analysis (disease progression, motor fluctuations, too extensive medication reduction with clinical worsening after surgery, etc.).

The CDRS change heatmap showed an antidyskinetic region in the central GPi, overlapping with antiparkinsonian as well as pro-parkinsonian areas in the off-period motor change map (Fig. 5). Notably, the antidyskinetic areas partially aligned with previously published antidystonic regions^19^ (Supplementary Fig. 8). The antidyskinetic effect of GPi-DBS supposedly has two components: (i) DBS can allow LED reduction and thus indirect improvement of dyskinesia; (ii) DBS may induce a direct antidyskinetic effect by stimulation itself in various indications.^8,32,33^ We assume—but cannot prove, based on our analysis—that stimulation of different fibre pathways originating within or passing through the pallidal region could underlie the spatially partly overlapping, yet functionally divergent effects.

The area related to worsening of dyskinesias was located dorsally, at the central GPi/GPe border and in the adjacent GPe, partially overlapping with the generally detrimental sourspot (Fig. 5). The dyskinesia worsening after dorsal stimulation is consistent with the results of Krack^13^ and Tsuboi *et al*.^16^

### Computer-assisted DBS programming

The coordinates of the sweetspot for off-period motor benefit may help to guide lead implantation. However, they are not sufficient to optimize programming settings at a given lead location; the variance explained by ACs distance from the sweetspot’s COG was dramatically lower than through the heatmap model (22% versus 72%). The probabilistic map suggests a more complex outcome distribution with several local maxima and minima, similar to our previous observation in pallidal DBS for dystonia,^19^ favouring more sophisticated, clinician-based anatomical or computer-assisted programming approaches.

Computer-assisted DBS programming aims not only to accelerate the programming procedure by navigating through the complex heatmap shape but also to eliminate unnecessary energy delivery, improve treatment outcomes, and help troubleshoot in non-responders. In patients with Parkinson’s disease treated with STN-DBS, an automated algorithm has recently been tested in a clinical trial, and the predicted parameters were shown to be non-inferior to the clinical choice.^34^ Reich *et al.*^19^ implemented digital optimization of stimulation parameters for dystonia patients treated with GPi-DBS. The optimization was based on a heatmap and a monopolar review in silico, inspiring our own work. Lange *et al.*^35^ tested the algorithm prospectively in a pilot study of ten patients, with superiority to clinical parameters. Rajamani *et al.*^2^ demonstrated symptom-specific digital reprogramming based on fibre-activation patterns in patients with Parkinson’s disease undergoing STN-DBS.

Here, we implemented an approach similar to Reich *et al.*^19^ in patients with Parkinson’s disease treated with GPi-DBS. We demonstrated a possible advantage of algorithm-derived programming choices over the clinical stimulation settings. Nevertheless, it is important to note that the optimized outcomes were predictions based on the overlap between simulated VTAs and our probabilistic outcome map, which must not be interpreted as true clinical outcomes. A clinical trial comparing algorithmic and clinical programming choices, however, might be justified based on our results. In addition to shortening the programming time and improving overall outcomes, the digital prediction of DBS settings may also be advantageous in patients with therapy failure. In our cohort, we found 11 patients (28%) with worsening of their Parkinson’s disease off-period motor symptoms after pallidal DBS. Our simulation suggested that only one of these patients had an implant failure, whereas in all other patients, optimized programming may have rescued the outcome (see Fig. 6). This patient may be a candidate for lead repositioning, as highlighted by a failure in ‘digital programming’.

Given the spatial complexities of the target region, reflected in the heatmap, and the advancing stimulation regimens with directional current steering and multiple current sources, sophisticated simulations exceeding the simple ‘monopolar review in silico’ will be needed to facilitate the clinical use of spatial maps. The development of such methods is a focus of current extensive research (e.g.^2,36^), although complicated by increasing computational expenses.

## Limitations

There are several methodological limitations specific to various steps of data processing and modelling that have been discussed in previous publications.^19,20,24,37^ Most importantly, the transformation into a common MNI space and the construction of a common map are ignorant of interindividual anatomical variability. The VTA models are oversimplified and the real stimulation effects of DBS on brain tissue are complex and non-binary. Nevertheless, these approaches are now well established, and an image-guided programming approach based on the VTA model has been successfully clinically employed.^35^ We tried to corroborate our results by using a different data processing toolbox^18^ and a different statistical pipeline depicted by Horn *et al.*,^24^ leading to an R-map that was predictive in an LOO fashion, similar to our model.

Next, the use of two different motor scales (MDS-UPDRS III and UPDRS III) might warrant some concerns. However, we consistently employ the percentage improvement from the baseline for our analyses, avoiding any direct comparison between incoherent scores. The use of motor scores and their relative postoperative reduction has several limitations per se, such as interrater variability or disease progression during the follow-up period. Nevertheless, this approach is well established in the field^2,14,19,37^ and has been clinically applied.^35^

Importantly, our statistical approach was not designed to draw any inferences at the voxel level. The spatial map was tested in several subsequent steps (LOO with 15 predictors, 9 predictors, LOOCV) within the framework of the predictive model, demonstrating predictiveness and generalizability of the aggregate map as a unit. This approach aligns with the supposition of a complex anatomical distribution and individualized programming, rather than hitting a single universal hotspot. In this concept, validation of the joint model replaces a traditional correction for multiple comparisons. However, the threshold of cluster volume > 50 voxels used to visualize the sweetspot and sourspot areas might be considered arbitrary in this context. Therefore, it is important to keep in mind that the sweetspots and sourpots illustrate the most important areas and serve as a visual representation of potential implantation targets, but the full map should always be considered.

Our study was performed retrospectively. This allowed correlative observations, but is not conclusive for causal relations, which would need to be evaluated in a prospective clinical trial design. The correlative conclusions, however, were validated using state-of-the-art statistical prediction methods, such as the LOOCV, simulating a prediction of unseen patients.

In addition, our methodology was purely topographical and did not include pathway analyses, which may be crucial for understanding anatomical-functional relations.^24,38,39^ As such, it more truthfully reflects the clinical reality of surgical planning and MRI-guided anatomical programming. Furthermore, our model did not integrate stimulation-induced adverse effects for optimizing stimulation volumes. However, the model was built on chronic VTAs, which implicitly include clinical optimization for adverse effects.

There are also multiple limitations to our subcohort analysis of the Amsterdam cohort. Only 23 patients were included (as the Shanghai patients lacked the dyskinesia scores), and we could not validate the heatmaps by demonstrating consistent predictiveness. Additionally, motor fluctuations and the pragmatic definition of on- and off-motor states could have influenced the UPDRS III and CDRS scoring, thus potentially creating a bias in the interindividual VTA comparisons. As discussed above, there were overlapping indirect and direct antidyskinetic effects within the final CDRS reduction with chronic pallidal DBS. These two aspects could not be differentiated using the present data. Moreover, the antidystonic map of Reich *et al.*^19^ was based on the global reduction in dystonia scores and did not distinguish between dystonic movements and postures—making it difficult to compare with the antidyskinetic map in Parkinson’s disease. Despite these caveats and the rather exploratory nature of this analysis, we believe that it could inspire further research and future attempts for individualized, symptom-oriented GPi-DBS programming based on more extensive cohorts (which would ideally include patients with directional leads), allowing for a more fine-graded probabilistic mapping.

In conclusion, the clinical outcome of GPi-DBS in our two independent cohorts with Parkinson’s disease was notably variable. A large portion of this variance could be explained by the topography of stimulation fields in the target area. We present a voxelwise probabilistic map of the DBS antiparkinsonian effect, describing in detail the GPi and its surrounding regions. This map could provide useful information for anatomical programming and lead implantation. The antiparkinsonian sweetspot lay at the anteromedial part of the ventrocaudal GPi; dorsal stimulation fields, including the GPe, were related to symptom worsening. The antidyskinetic areas were located within the central GPi and partially overlapped with antiparkinsonian regions. In the future, our heatmap could be applied to digitally optimize stimulation parameters and eventually reduce the outcome variability of pallidal DBS in Parkinson’s disease.

## Supplementary Material

fcaf374_Supplementary_Data

## Data Availability

The imaging data cannot be provided online for legal reasons and will be shared upon a reasonable request. The code and the spatial maps are available as part of the Arena toolbox under https://github.com/visualDBSlab/ARENA. The clinical data are provided in the supplementary materials.

## References

[1] Lozano AM, Lang AE, Galvez-Jimenez N, et al Effect of GPi pallidotomy on motor function in Parkinson's disease. Lancet. 1995;346(8987):1383–1387. 7475819 10.1016/s0140-6736(95)92404-3

[2] Rajamani N, Friedrich H, Butenko K, et al Deep brain stimulation of symptom-specific networks in Parkinson's disease. Nat Commun. 2024;15(1):4662. 38821913 10.1038/s41467-024-48731-1 PMC11143329

[3] Peng L, Fu J, Ming Y, Zeng S, He H, Chen L. The long-term efficacy of STN vs GPi deep brain stimulation for Parkinson disease: A meta-analysis. Medicine (Baltimore). 2018;97(35):e12153. 30170458 10.1097/MD.0000000000012153 PMC6393030

[4] Odekerken VJ, van Laar T, Staal MJ, et al Subthalamic nucleus versus globus pallidus bilateral deep brain stimulation for advanced Parkinson's disease (NSTAPS study): A randomised controlled trial. Lancet Neurol. 2013;12(1):37–44. 23168021 10.1016/S1474-4422(12)70264-8

[5] Deuschl G, Antonini A, Costa J, et al European academy of neurology/movement disorder society-European section guideline on the treatment of Parkinson's disease: I. Invasive therapies. Mov Disord. 2022;37(7):1360–1374. 35791767 10.1002/mds.29066

[6] Follett KA, Weaver FM, Stern M, et al Pallidal versus subthalamic deep-brain stimulation for Parkinson's disease. N Engl J Med. 2010;362(22):2077–2091. 20519680 10.1056/NEJMoa0907083

[7] Xu H, Zheng F, Krischek B, et al Subthalamic nucleus and globus pallidus internus stimulation for the treatment of Parkinson's disease: A systematic review. J Int Med Res. 2017;45(5):1602–1612. 28701061 10.1177/0300060517708102 PMC5718722

[8] Boogers A, Fasano A. A transatlantic viewpoint on the role of pallidal stimulation for Parkinson's disease. Mov Disord. 2024;39(1):36–39. 37965914 10.1002/mds.29656

[9] Combs HL, Folley BS, Berry DT, et al Cognition and depression following deep brain stimulation of the subthalamic nucleus and globus Pallidus pars internus in Parkinson's disease: A meta-analysis. Neuropsychol Rev. 2015;25(4):439–454. 26459361 10.1007/s11065-015-9302-0

[10] Tsuboi T, Lopes JLMLJ, Patel B, et al Parkinson's disease motor subtypes and bilateral GPi deep brain stimulation: One-year outcomes. Parkinsonism Relat Disord. 2020;75:7–13. 32428801 10.1016/j.parkreldis.2020.05.004

[11] Sobesky L, Goede L, Odekerken VJJ, et al Subthalamic and pallidal deep brain stimulation: Are we modulating the same network? Brain. 2022;145(1):251–262. 34453827 10.1093/brain/awab258

[12] Bejjani B, Damier P, Arnulf I, et al Pallidal stimulation for Parkinson's disease. Two targets? Neurology. 1997;49(6):1564–1569. 9409347 10.1212/wnl.49.6.1564

[13] Krack P, Pollak P, Limousin P, et al Opposite motor effects of pallidal stimulation in Parkinson's disease. Ann Neurol. 1998;43(2):180–192. 9485059 10.1002/ana.410430208

[14] Elias GJB, Boutet A, Joel SE, et al Probabilistic mapping of deep brain stimulation: Insights from 15 years of therapy. Ann Neurol. 2021;89(3):426–443. 33252146 10.1002/ana.25975

[15] Middlebrooks EH, Tuna IS, Grewal SS, et al Segmentation of the globus Pallidus internus using probabilistic diffusion tractography for deep brain stimulation targeting in Parkinson disease. AJNR Am J Neuroradiol. 2018;39(6):1127–1134. 29700048 10.3174/ajnr.A5641 PMC7410622

[16] Tsuboi T, Charbel M, Peterside DT, et al Pallidal connectivity profiling of stimulation-induced dyskinesia in Parkinson's disease. Mov Disord. 2021;36(2):380–388. 33002233 10.1002/mds.28324

[17] Wong JK, Hilliard JD, Holanda VM, et al Time for a new 3-D image for globus Pallidus internus deep brain stimulation targeting and programming. J Parkinsons Dis. 2021;11(4):1881–1885. 34420982 10.3233/JPD-212820 PMC8609712

[18] Horn A, Li N, Dembek TA, et al Lead-DBS v2: Towards a comprehensive pipeline for deep brain stimulation imaging. Neuroimage. 2019;184:293–316. 30179717 10.1016/j.neuroimage.2018.08.068 PMC6286150

[19] Reich MM, Horn A, Lange F, et al Probabilistic mapping of the antidystonic effect of pallidal neurostimulation: A multicentre imaging study. Brain. 2019;142(5):1386–1398. 30851091 10.1093/brain/awz046

[20] Astrom M, Diczfalusy E, Martens H, Wardell K. Relationship between neural activation and electric field distribution during deep brain stimulation. IEEE Trans Biomed Eng. 2015;62(2):664–672. 25350910 10.1109/TBME.2014.2363494

[21] Fonov V, Evans AC, Botteron K, et al Unbiased average age-appropriate atlases for pediatric studies. Neuroimage. 2011;54(1):313–327. 20656036 10.1016/j.neuroimage.2010.07.033 PMC2962759

[22] Ewert S, Plettig P, Li N, et al Toward defining deep brain stimulation targets in MNI space: A subcortical atlas based on multimodal MRI, histology and structural connectivity. Neuroimage. 2018;170:271–282. 28536045 10.1016/j.neuroimage.2017.05.015

[23] Treu S, Strange B, Oxenford S, et al Deep brain stimulation: Imaging on a group level. Neuroimage. 2020;219:117018. 32505698 10.1016/j.neuroimage.2020.117018

[24] Horn A, Reich MM, Ewert S, et al Optimal deep brain stimulation sites and networks for cervical vs. Generalized dystonia. Proc Natl Acad Sci U S A. 2022;119(14):e2114985119. 35357970 10.1073/pnas.2114985119 PMC9168456

[25] Okun MS, Foote KD. Subthalamic nucleus vs globus pallidus interna deep brain stimulation, the rematch: Will pallidal deep brain stimulation make a triumphant return? Arch Neurol. 2005;62(4):533–536. 15824249 10.1001/archneur.62.4.533

[26] Volkmann J, Moro E, Pahwa R. Basic algorithms for the programming of deep brain stimulation in Parkinson's disease. Mov Disord. 2006;21(Suppl 14):S284–S289. 16810675 10.1002/mds.20961

[27] Koeglsperger T, Palleis C, Hell F, Mehrkens JH, Botzel K. Deep brain stimulation programming for movement disorders: Current concepts and evidence-based strategies. Front Neurol. 2019;10:410. 31231293 10.3389/fneur.2019.00410 PMC6558426

[28] Au KLK, Wong JK, Tsuboi T, et al Globus Pallidus internus (GPi) deep brain stimulation for Parkinson's disease: Expert review and commentary. Neurol Ther. 2021;10(1):7–30. 33140286 10.1007/s40120-020-00220-5 PMC8140010

[29] Tisch S, Zrinzo L, Limousin P, et al Effect of electrode contact location on clinical efficacy of pallidal deep brain stimulation in primary generalised dystonia. J Neurol Neurosurg Psychiatry. 2007;78(12):1314–1319. 17442760 10.1136/jnnp.2006.109694 PMC2095629

[30] da Silva Lapa JD, Godinho FLF, Teixeira MJ, et al Should the globus Pallidus targeting be refined in dystonia? J Neurol Surg A Cent Eur Neurosurg. 2022;83(4):361–367. 34808675 10.1055/s-0041-1735856

[31] Fleury V, Pollak P, Gere J, et al Subthalamic stimulation may inhibit the beneficial effects of levodopa on akinesia and gait. Mov Disord. 2016;31(9):1389–1397. 26887333 10.1002/mds.26545

[32] Smith KM, Spindler MA. Uncommon applications of deep brain stimulation in hyperkinetic movement disorders. Tremor Other Hyperkinet Mov (N Y). 2015;5:278. 25713746 10.7916/D84X56HP PMC4314611

[33] Koy A, Kuhn AA, Schiller P, et al Long-term follow-up of pediatric patients with dyskinetic cerebral palsy and deep brain stimulation. Mov Disord. 2023;38(9):1736–1742. 37358761 10.1002/mds.29516

[34] Roediger J, Dembek TA, Achtzehn J, et al Automated deep brain stimulation programming based on electrode location: A randomised, crossover trial using a data-driven algorithm. Lancet Digit Health. 2023;5(2):e59–e70. 36528541 10.1016/S2589-7500(22)00214-X

[35] Lange F, Soares C, Roothans J, et al Machine versus physician-based programming of deep brain stimulation in isolated dystonia: A feasibility study. Brain Stimul. 2023;16(4):1105–1111. 37422109 10.1016/j.brs.2023.06.018

[36] Malekmohammadi M, Mustakos R, Sheth S, et al Automated optimization of deep brain stimulation parameters for modulating neuroimaging-based targets. J Neural Eng. 2022;19(4):046014. 10.1088/1741-2552/ac7e6c PMC11090244 35790135

[37] Dembek TA, Baldermann JC, Petry-Schmelzer J-N, et al Sweetspot mapping in deep brain stimulation: Strengths and limitations of current approaches. Neuromodulation. 2022;25(6):877–887. 33476474 10.1111/ner.13356

[38] Hollunder B, Ostrem JL, Sahin IA, et al Mapping dysfunctional circuits in the frontal Cortex using deep brain stimulation. Nat Neurosci. 2024;27(3):573–586. 38388734 10.1038/s41593-024-01570-1 PMC10917675

[39] Butenko K, Li N, Neudorfer C, et al Linking profiles of pathway activation with clinical motor improvements—A retrospective computational study. Neuroimage Clin. 2022;36:103185. 36099807 10.1016/j.nicl.2022.103185 PMC9474565

