# Supplementary material for: Probabilistic mapping of the antiparkinsonian effects of pallidal deep brain stimulation
Source: Brain Commun. 2025 Sep 26;7(5):fcaf374. doi: 10.1093/braincomms/fcaf374 (PMC12516314; doi:10.1093/braincomms/fcaf374)
Supplement: fcaf374_Supplementary_Data [file fcaf374_supplementary_data.pdf]

Supplementary Table 1: Cohort summary.

ID	Sex	Age at surgery	Baseline MED OFF motor scores*	Post-op MED OFF motor scores*	Follow-up (moths postop)	Lead	Stimulation parameter: left					Stimulation parameter: right					Outcomes			Optimal contacts		Optimal amplitudes		LED_% change	UPDRS-III MED ON_% change	CDRS_% change
							frequency	pulse width	voltag e	active contacts	grounding	frequency	pulse width	voltage	active contacts	grounding	empirical	predicted	optimized (12345mA)	left	right	left	right			
ZZ01_Sha	w	69	0.87	0.88	12	Medtronic 3387	130	60	2.1	0 1	case	130	60	2.3	0 1	case	0	0.11	0.88	0	3	2	1			
ZZ02_Sha	w	70	1.24	1.30	12	Medtronic 3387	145	70	3.7	2 3	case	145	60	3.3	2 3	case	-0.05	0.16	0.44	3	0	2	1			
ZZ03_Sha	m	69	3.09	1.70	12	Medtronic 3387	160	60	2.75	1	case	160	60	3.35	0 1	case	0.45	0.71	0.35	1	1	1	1			
ZZ07_Sha	w	67	1	0.67	8	SceneRay 1210	115	90	3.3	0 2	case	105	90	3.3	0 2	case	0.33	0.37	1.03	1	0	2	2			
ZZ13_Sha	m	68	1.72	1.42	17	PINS L302	60	90	3.25	1 2	case	60	120	3.35	1 2	case	0.18	0.12	0.58	2	3	1	1			
ZZ14_Sha	w	54	1.51	1.18	12	Medtronic 3387	145	60	2.45	0 1	case	145	60	2.75	2 3	case	0.04	0.05	0.45	2	3	1	1			
ZZ16_Sha	m	66	1.75	1.21	15	Medtronic 3387	145	80	4	1 2	case	145	90	3.75	1 2	case	0.31	0.31	0.52	1	0	1	1			
ZZ17_Sha	m	69	1.15	1.33	15	Medtronic 3387	60	160	3.3	2 3	case	60	160	3	2 3	case	-0.16	0.07	0.53	0	2	1	1			
ZZ18_Sha	w	64	1.78	1.33	10	Medtronic 3387	145	70	3.45	0 1	case	145	70	2.85	1 3	case	0.25	0.25	0.47	1	1	1	5			
ZZ20_Sha	m	52	1.66	1.36	6	Medtronic 3387	160	60	3.25	1 3	case	160	70	3	2 3	case	0.18	0.38	0.75	3	0	1	2			
ZZ21_Sha	m	60	1.15	1.64	28	Medtronic 3387	70	180	2.95	0	case	60	180	3.15	1 3	case	-0.42	-0.40	0.47	2	1	1	1			
ZZ23_Sha	w	61	2	0.58	12	Medtronic 3387	145	70	3.5	0 1	case	145	60	3.5	1 2	case	0.65	0.59	0.65	1	0	1	3			
ZZ27_Sha	w	65	1.78	1.73	12	Medtronic 3387	160	60	3.5	2 3	case	160	60	3.25	2 3	case	0.03	0.01	0.22	1	3	5	5			
ZZ28_Sha	m	65	1.63	0.52	9	Medtronic 3387	80	125	3.45	1	case	80	125	3.2	2	case	0.69	0.71	0.64	3	2	2	1			
ZZ29_Sha	m	71	0.81	1.33	18	Medtronic 3387	60	160	2.5	1 2	case	60	160	3.5	2 3	case	-0.63	-0.34	1.04	2	0	1	3			
ZZ33_Sha	m	53	1.87	1.30	9	Medtronic 3387	135	70	3.25	1 3	case	160	70	3	2 3	case	0.31	0.30	0.31	2	3	1	1			
ZZ40_AMS	m	61	1.85	0.52	12	Medtronic 3389	130	90	2.4	1	case	130	90	2.3	1	case	0.72	0.39	0.26	3	0	1	1	0.4333	0.67549	-1
ZZ41_AMS	m	57	1.59	1.85	12	Medtronic 3389	130	60	2	1	case	130	60	2	2 1 0	case	-0.16	0.02	1.02	3	2	2	1	-0.0656	0.10478	0
ZZ43_AMS	w	67	0.89	1.33	12	Medtronic 3389	185	60	2.8	2 1 0	case	185	60	2.3	2 1 0	case	-0.5	-0.40	0.14	0	0	1	2	0.5414	-0.73077	0.71429
ZZ44_AMS	m	50	1.22	1.26	12	Medtronic 3389	130	120	3.3	1	case	130	90	1.8	1 0	case	-0.03	0.25	0.83	1	0	5	1	0.2105	0.13461	0.375
ZZ45_AMS	m	62	1.07	1.70	12	Medtronic 3389	130	60	3	2	case	130	60	3.5	1 0	2	-0.59	-0.09	0.51	3	2	1	1	0.2308	-0.38462	0.33333
ZZ46_AMS	m	69	1.70	1.30	12	Medtronic 3389	185	90	3.5	2 1	case	130	60	3.3	2 1	case	0.24	-0.01	0.14	1	3	1	3	0.5283	-0.77149	1
ZZ48_AMS	m	72	1.63	0.63	12	Medtronic 3389	130	60	3.5	2	case	30	60	3.5	2	case	0.61	0.25	0.40	0	2	1	1	0.2339	0.32805	1
ZZ49_AMS	w	61	1.15	1.15	12	Medtronic 3389	130	90	3.3	2 1	case	130	60	3.2	2 1	case	0	-0.21	0.15	3	1	2	5	-0.0508	-0.5	-0.0833
ZZ50_AMS	m	59	2.59	1.59	12	Medtronic 3389	130	60	3.3	1	case	130	60	3.3	1	case	0.39	0.34	-0.46	3	2	1	3	0.1379	0.2730769	1
ZZ51_AMS	w	65	1.15	0.63	12	Medtronic 3389	130	60	3.5	2	case	130	60	3.5	1	case	0.45	0.11	0.58	1	0	1	1	0.1758	0.65384	-0.1429

ZZ53_AMS	m	60	2.07	1.30	12	Medtronic 3389	130	60	3.3	2	case	130	60	2.8	3	case	0.38	0.28	0.55	3	3	3	1	-0.096	0.10989	0.5
ZZ54_AMS	m	62	2.48	1.37	12	Medtronic 3389	130	60	2.8	1	case	130	60	3.3	1	case	0.45	0.44	0.66	0	3	1	1	0.125	0.20588	1
ZZ55_AMS	w	60	2.44	1.52	12	Medtronic 3389	130	60	2	2	case	130	60	3.3	1	case	0.38	0.32	0.98	3	2	2	2	0.5455	-0.76538	1
ZZ56_AMS	m	58	1.22	1.89	12	Medtronic 3389	30	60	2	2 1	case	130	60	2	1	case	-0.55	-0.24	0.44	2	3	1	1	0.5594	-0.3664	1
ZZ57_AMS	m	53	1.56	1.04	12	Medtronic 3389	130	60	3	1	case	130	60	2.5	2 1	case	0.33	0.33	0.33	2	1	1	1	-0.183	-0.47115	0
ZZ58_AMS	w	64	0.89	0.37	12	Medtronic 3389	130	60	3.5	0	case	130	60	3.5	0	case	0.58	0.40	0.30	2	2	1	2	0.2857	1	1
ZZ60_AMS	w	50	1.70	2.00	12	Medtronic 3389	130	60	3.5	1	case	130	60	3.5	0	case	-0.17	-0.24	0.09	2	2	1	1	0.3271	-0.55769	-1
ZZ61_AMS	w	65	1.52	1.04	12	Medtronic 3389	130	60	3.5	1	case	130	60	3	1	case	0.32	0.17	0.82	1	1	5	1	0.431	0.48077	0.66667
ZZ62_AMS	m	46	0.96	1.37	12	Medtronic 3389	130	60	3.3	1 0	case	130	60	3.3	1 0	case	-0.42	-0.77	0.47	3	3	1	1	-0.0263	-1.76923	0.92308
ZZ63_AMS	m	62	1.85	1.19	12	Medtronic 3389	130	60	3	2 1	case	130	60	3.2	2 1	case	0.36	0.30	0.33	0	1	1	1	0.2079	0.53846	0.75
ZZ64_AMS	m	55	1.44	0.89	12	Medtronic 3389	130	60	1.5	3	case	130	60	2.5	3	case	0.38	0.23	0.31	3	0	1	1	0.0494	0.13461	0.66667
ZZ65_AMS	m	60	2.26	1.22	12	Medtronic 3389	130	60	3.3	1	case	185	150	3.5	2	case	0.46	0.52	0.47	3	1	4	5	0	-0.11834	1
ZZ66_AMS	w	62	2.07	1.33	12	Medtronic 3389	130	60	2.5	1	case	130	60	2.3	1	case	0.36	0.33	0.68	3	0	2	3	-0.06	-0.11538	1

*Normalized

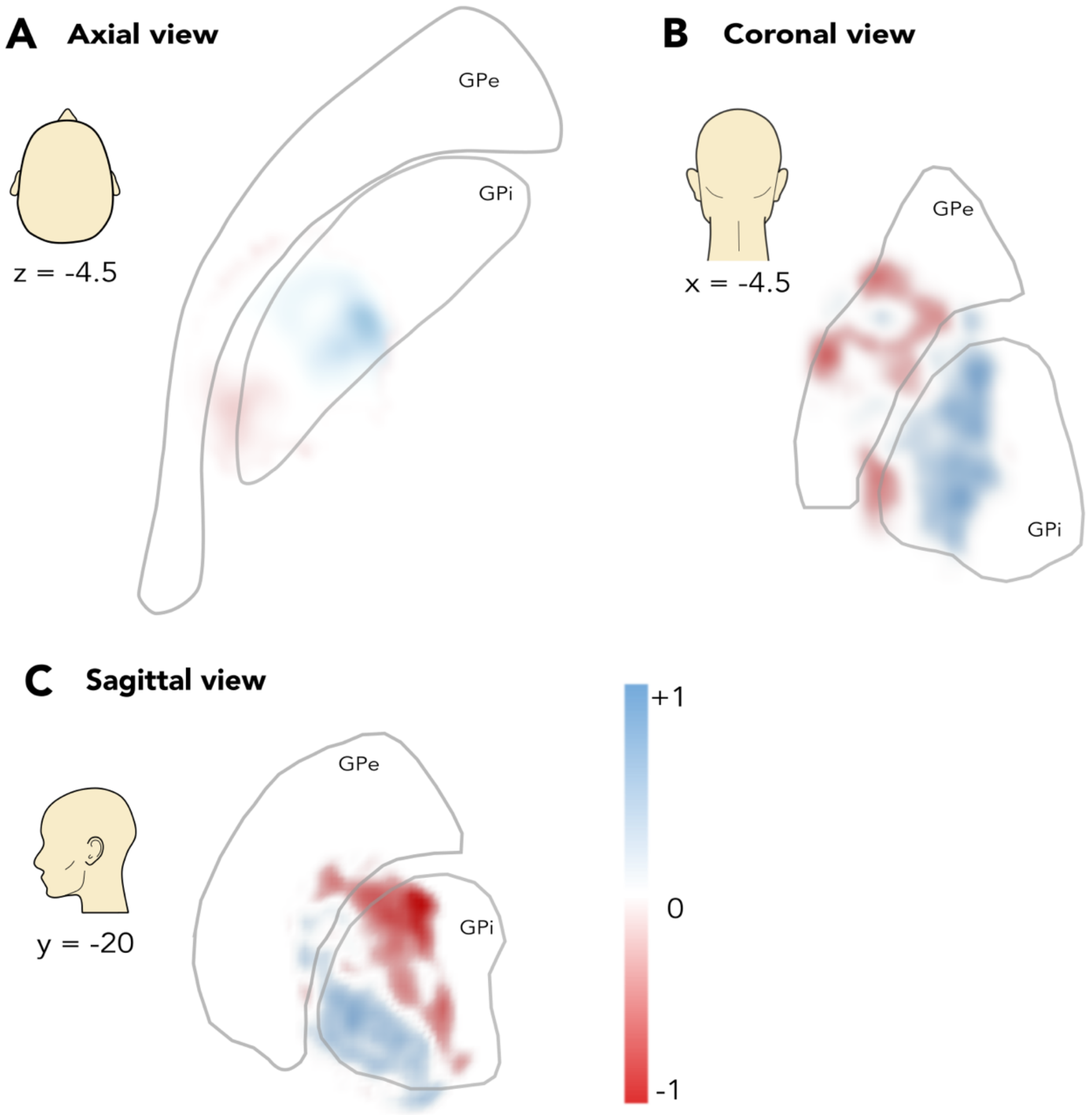

Supplementary Figure 1: The R-map obtained from lead group analysis[1] *(n=39) in axial (A), coronal (B), and sagittal slices (C) with the pallidal complex. Spearman´s rank correlation between E-field magnitudes and clinical outcomes was calculated in each voxel. Detrimental voxels are depicted in tones of red, and beneficial voxels are in tones of blue.*

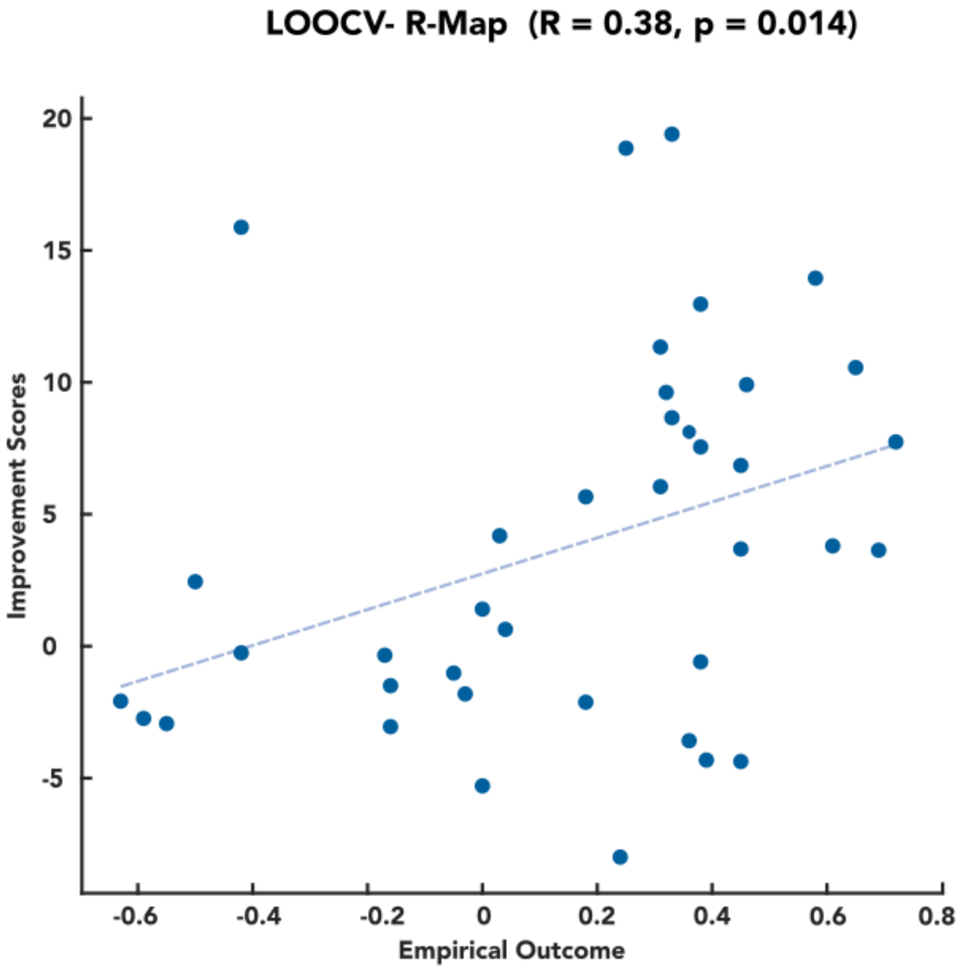

Supplementary Figure 2: The R-map constructed according to Horn et al[2] *performs well in leave-one-out cross-validation, Pearson´s R = 0.38, p = 0.014. Datapoints correspond to individual subjects.*

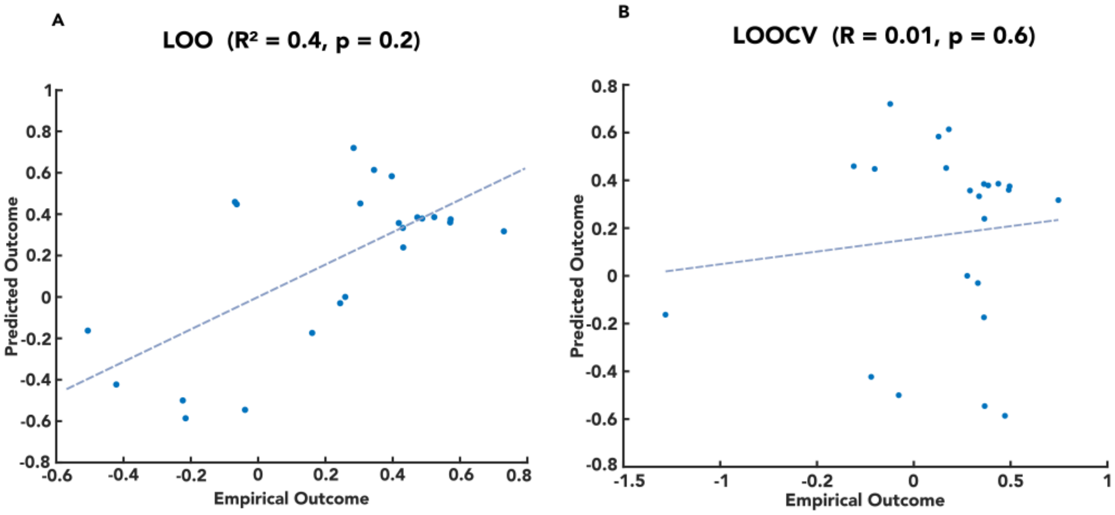

Supplementary Figure 3: Prediction of UPDRS-III Percentage Reduction in MED OFF (Amsterdam Cohort): A multivariate linear model with 9 bin interference histograms acquired in leave-one-out settings as input (A), the F-test reveals a non-significant p-value of $p = 0.2$. Simulating a prediction of an unseen patient in leave-one-out cross-validation yielded a Pearson´s correlation of empirical and predicted outcomes with $R = 0.01$, $p = 0.6$ (B). Datapoints correspond to individual subjects.

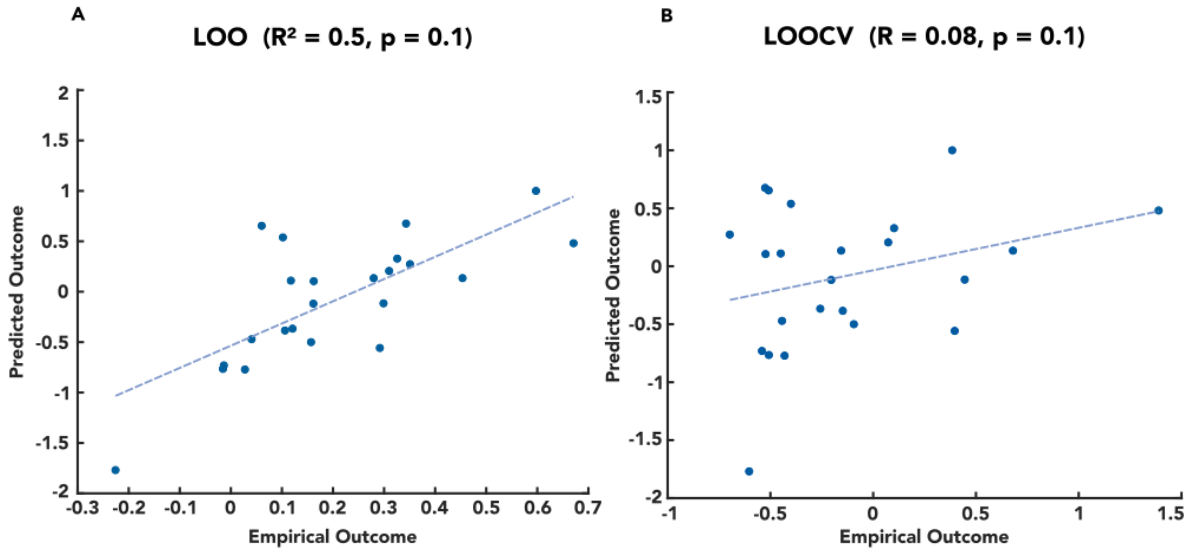

Supplementary Figure 4: Prediction of UPDRS-III Percentage Reduction in MED ON: A multivariate linear model with 9 bin interference histograms acquired in leave-one-out settings as input (A), the F-test reveals a non-significant p-value of p = 0.1. Simulating a prediction of an unseen patient in leave-one-out cross-validation yielded a Pearson´s correlation of empirical and predicted outcomes with R = 0.08, p = 0.1 (B). Datapoints correspond to individual subjects.

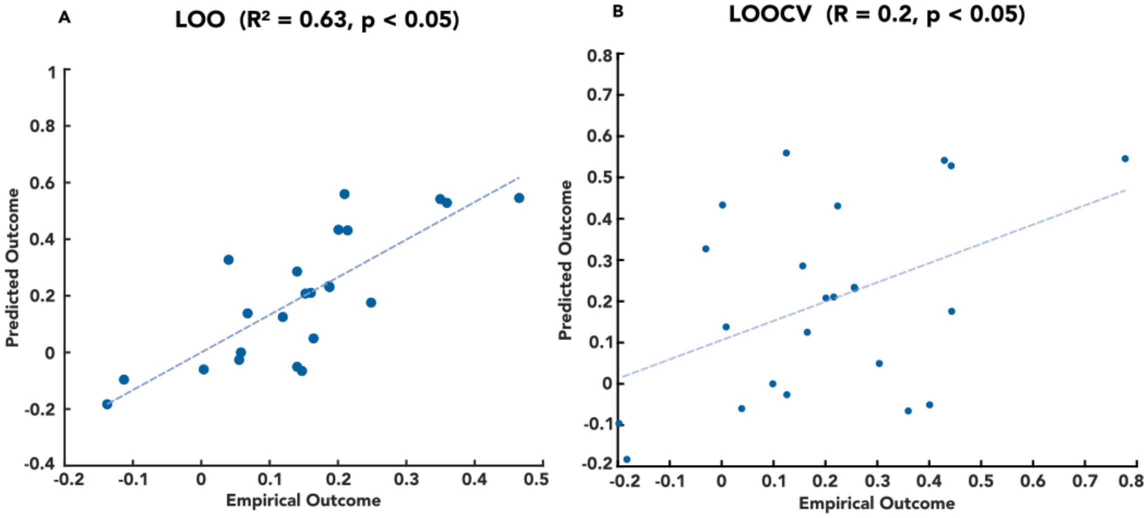

Supplementary Figure 5: Prediction of the LED Percentage Reduction: The multivariate linear model with interference histograms acquired in leave-one-out settings as input explains 63% of the outcome variance (A), the F-test reveals significance of $p < 0.05$. Simulating a prediction of an unseen patient in leave-one-out cross-validation yielded a Pearson´s correlation of empirical and predicted outcomes with $R = 0.2$, $p < 0.05$ (B). Datapoints correspond to individual subjects.

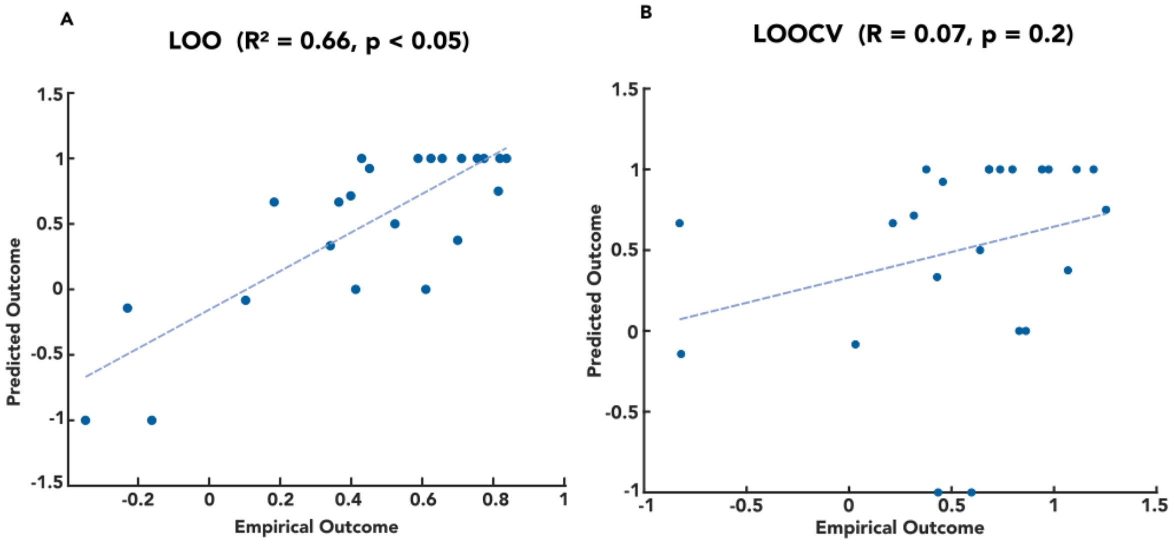

Supplementary Figure 6: Prediction of the CDRS Percentage Reduction: The multivariate linear model with interference histograms acquired in leave-one-out settings as input explains 66% of the outcome variance (A), the F-test reveals significance of $p < 0.05$. Simulating a prediction of an unseen patient in leave-one-out cross-validation yielded a Pearson´s correlation of empirical and predicted outcomes with $R = 0.07$, $p = 0.2$ (B). Datapoints correspond to individual subjects.

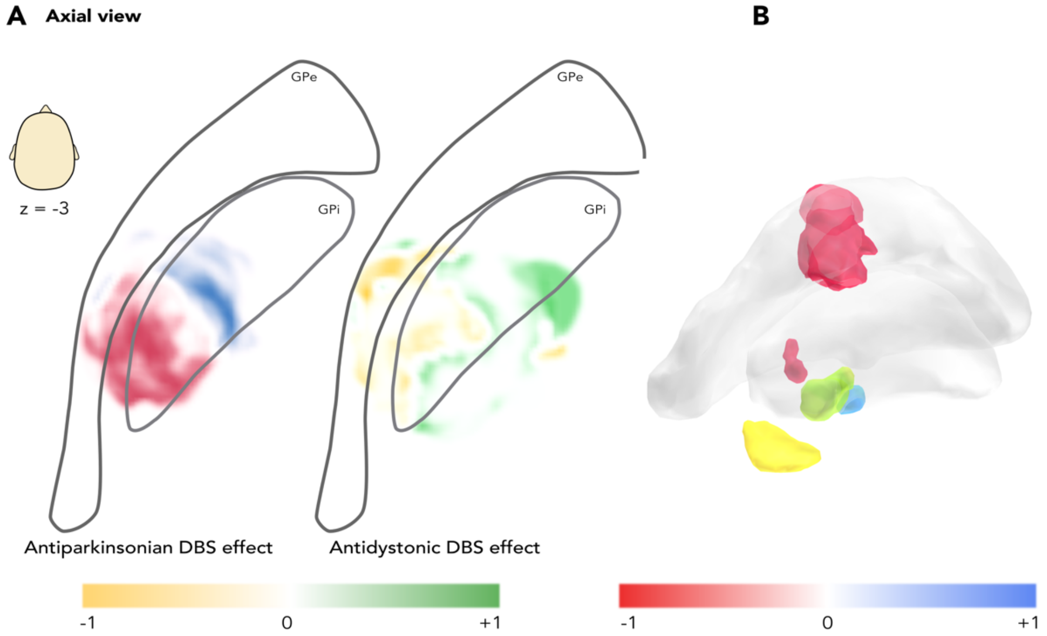

Supplementary Figure 7: (A) The heatmap of antiparkinsonian (blue and red) DBS effect (n=39, see also the main Figure 3) with the antidystonic heatmap from Reich et al.[3] *(yellow and green, n=87) in axial slices. Detrimental voxels are depicted in tones of red/yellow, and beneficial voxels are in tones of blue/green within the pallidal complex. Note the perpendicular gradient of the heatmaps. (B) The most significant voxel clusters form sweetspots and sourspots from both maps. In contrast to sourspots, the antidystonic and antiparkinsonian sweetspots lie in a tight vicinity in the ventral GPi.*

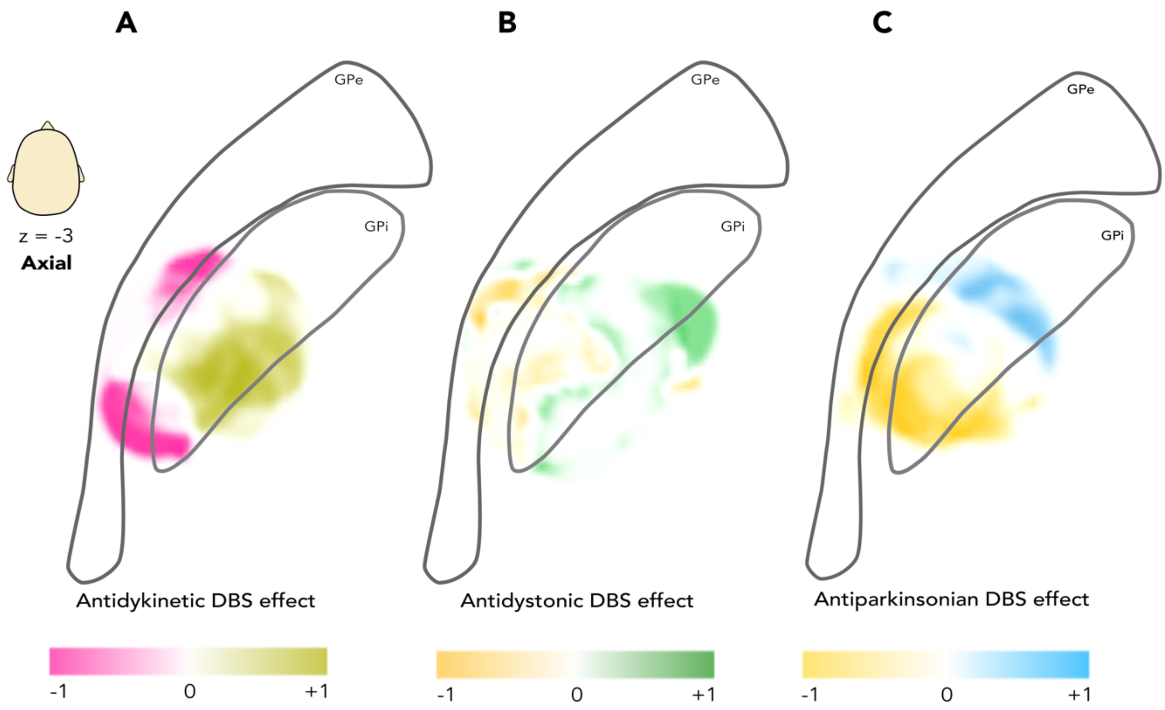

Supplementary Figure 8: (A) The Antidyskinetic Heatmap from Amsterdam sub-cohort (n=23, see also the main Figure 5), (B) The Antidystonic Heatmap from Reich et al.[3] *(n=87), (C) The Antiparkinsonian Heatmap from Amsterdam sub-cohort (n=23) in axial slices with the pallidal complex.*

Supplementary references:

1. *Treu S, Strange B, Oxenford S, et al. Deep brain stimulation: Imaging on a group level. Neuroimage. 2020;219:117018. doi:10.1016/j.neuroimage.2020.117018*
2. *Horn A, Reich MM, Ewert S, et al. Optimal deep brain stimulation sites and networks for cervical vs. generalized dystonia. Proc Natl Acad Sci U S A. 2022;119(14):e2114985119. doi:10.1073/pnas.2114985119*
3. *Reich MM, Horn A, Lange F, et al. Probabilistic mapping of the antidystonic effect of pallidal neurostimulation: a multicentre imaging study. Brain. 2019;142(5):1386-1398. doi:10.1093/brain/awz046*
